# Optimizing culture-free approaches to recover high-quality Mycobacterium tuberculosis genomic variation

**DOI:** 10.1099/mgen.0.001806

**Published:** 2026-08-18

**Authors:** Katharine S. Walter, Paulo César Pereira Dos Santos, Allison Carey, Salika M. Shakir, Caroline Colijn, Ted Cohen, Barun Mathema, Julio Croda, Jason R. Andrews

**Affiliations:** 1 Division of Epidemiology, University of Utah, Salt Lake City, UT, USA; 2 College of Medicine, Federal University of Mato Grosso do Sul, Campo Grande, Brazil; 3 Department of Pathology, University of Utah, Salt Lake City, UT, USA; 4 ARUP Institute for Research and Innovation, Salt Lake City, UT, USA; 5 Department of Mathematics, Simon Fraser University, Burnaby, Canada; 6 Department of Epidemiology of Microbial Diseases, Yale School of Public Health, New Haven, CT, USA; 7 Department of Epidemiology, Columbia University Mailman School of Public Health, New York, NY, USA; 8 Mato Grosso do Sul Office, Oswaldo Cruz Foundation, Campo Grande, Brazil; 9 Division of Infectious Diseases and Geographic Medicine, Stanford University School of Medicine, Stanford, CA, USA

**Keywords:** genomics, *Mycobacterium tuberculosis*, hybrid capture, variant identification

## Abstract

**Background.** *Mycobacterium tuberculosis* (*Mtb*) genomic epidemiology often relies on culturing patient sputum, a time- and labour-intensive process. Hybrid capture approaches have been successfully used to enrich *Mtb* DNA from complex clinical samples, yet the accuracy of variant identification from captured samples has not been systematically evaluated.

**Methods.** We created artificial strain mixtures of two well-characterized *Mtb* isolates such that the minor strain comprised 0–50% of *Mtb* DNA and serially diluted the *Mtb* DNA into human DNA to simulate diagnostic samples with different sputum smear burdens (32 samples). We also prospectively collected paired *Mtb* diagnostic cultures and sputum submitted to a national diagnostic laboratory (seven sample pairs). We performed hybrid capture and Illumina whole-genome sequencing for all samples. For the artificial strain mixtures, we measured hybrid capture efficiency, the percentage of total reads mapping to *Mtb* and performance of fixed and minority variant identification. For the diagnostic samples, we compared the number, identity and minor allele frequencies of minority variants identified in the cultured and hybrid-captured samples.

**Results.** In the artificial strain mixture experiment, hybrid capture efficiency was 97% when *Mtb* comprised 0.01% of the input DNA. SNP identification via hybrid capture had a sensitivity ≥91% and precision ≥97% for *Mtb* lineages 4.1.2.1 and 4.9, excluding PE/PPE genes, when *Mtb* comprised 0.01% of input DNA. Observed minor allele frequencies were closely correlated (*r*=0.62 to *r*=0.79, *P*<0.001) with input minor allele frequencies across all dilutions. Among paired diagnostic samples, hybrid capture efficiency was high, 95%. However, four of the seven captured sputum samples were overwhelmed with *Pseudomonas* contamination, which comprised >25% of sequence reads. We did not detect a significant difference in the number of minority variants identified in cultured (median, 14; range, 10–18) and hybrid-captured samples (median: 17 variants, range 9–47, *P*=0.25) and minor allele frequencies were correlated (*r*=0.87, *P*<0.001) outside of PE/PPE genes.

**Conclusions.** Hybrid capture of diagnostic sputum samples efficiently recovers accurate *Mtb* whole-genome sequences. Minority variant detection is robust in controlled mixtures but shows reduced concordance in clinical samples, highlighting the need for further validation in real-world settings.

**Impact Statement**

*Mycobacterium tuberculosis* (*Mtb*) genomic epidemiology often relies on culturing patient sputa, which is time- and labour-intensive. Hybrid capture approaches enrich *Mtb* DNA from complex clinical samples, yet the efficiency and accuracy of identification of both consensus and minority variants from captured samples has not been systematically evaluated. To address these questions, we assessed the accuracy of hybrid capture both in experimental strain mixtures of well-characterized *Mtb* isolates and in clinical diagnostic samples. We found that hybrid capture of sputa samples can be used to efficiently sequence *Mtb* DNA from complex mixtures and that variant identification is highly accurate. Our results suggest that hybrid capture may offer an alternative to culture-based sequencing that could extend the coverage of genomic epidemiology studies.

## Data Summary

Sequence data for all samples are available on the Sequence Read Archive, as part of PRJNA1390833. Individual sample accession numbers are listed in Table S1.

## Introduction

Genomic epidemiology is a powerful tool for understanding *Mycobacterium tuberculosis* (*Mtb*) transmission patterns, evolutionary processes and the mechanisms of antibiotic resistance [1]. Current routine approaches for *Mtb* genomic epidemiology rely on culturing patient sputum to generate sufficient *Mtb* DNA for sequencing [1]. Mycobacterial culture is time- and labour-intensive and requires laboratory biosafety level 3 infrastructure. Culture may also impose a bottleneck on *Mtb* variation, as *Mtb* diversity present in sputa may be lost or outcompeted in culture, and some strains are not successfully isolated in culture [2–4]. However, a recent study has reported no loss of *Mtb* variation attributable to bacterial culture [5]. Further, producing sputum is difficult for many patients, including children, HIV-positive individuals and asymptomatic individuals [6]. While other clinical specimen types can be used for culture, this is not routinely done, with the result that many individuals are excluded from *Mtb* genomic studies.

Several approaches have been proposed to bypass culture and enrich *Mtb* DNA or deplete human DNA, enabling direct sequencing from clinical samples. This is necessary because *Mtb* DNA often comprises a small fraction of the total DNA [7, 8]. Metagenomic or shotgun sequencing – in which total sample DNA is sequenced – generates sequence reads that are primarily non-*Mtb*-derived and do not result in sufficient coverage for phylogenetic and transmission inference nor genotyping of antimicrobial resistance [7]. Several approaches for eukaryotic DNA depletion, often with detergents, have increased the efficiency of metagenomic sequencing of *Mycobacterium abscessus* and other respiratory pathogens [9, 10]. One study evaluated different methods for reducing levels of host DNA and found that benzonase treatment successfully enriched *Mtb* DNA as compared to host DNA [11]. Hybrid capture, the selective enrichment of target DNA via hybridization with complementary RNA probes, has enabled the *Mtb* sequencing directly from sputum samples [5, 8, 12–14]. Culture has previously been reported to act as a bottleneck on *Mtb* diversity present in a clinical sample [15]; however, a recent study reported that genetic diversity within hybrid-captured sputum mirrors that in matched *Mtb* cultures and highlighted the importance of appropriate variant filtering [5]. More recently, a tiled amplicon sequencing approach, based on the widely used approach for sequencing severe acute respiratory syndrome coronavirus 2 and other viruses, has been adapted for *Mtb* [16].

Previous studies have demonstrated that hybrid capture enables sequencing of *Mtb* DNA from complex clinical samples, enabling transmission inference and genotypic prediction of antimicrobial resistance [8, 12–14]. Hybrid capture has also been used successfully to sequence *Mtb* DNA from environmental samples [17]. Yet, the accuracy of variant identification from hybrid-captured samples has not been systematically evaluated, and whether variant accuracy varies with *Mtb* genomic background is unknown. Further, whether there is a limit of detection, a minimum sample proportion of *Mtb* DNA required for successful hybrid capture has not been measured.

Within-host *Mtb* genomic variation is of increasing interest clinically and epidemiologically [18–20]. Such within-host variation, sometimes referred to as minority variation when the *Mtb* population contains unfixed, or minority variants, which comprise less than 50% of the total population, can reflect a mixed or polyclonal infection of two distinct *Mtb* strains, or it can reflect *de novo* variation generated through mutations occurring over the course of a single infection. As with fixed variants, the accuracy of minority variants identified via hybrid capture has not yet been evaluated. Previous studies have been inconclusive about the impact of hybrid capture on detection of minority variation [8, 15]. For example, one previous study found no difference between the minority variation detected in cultured and hybrid-captured sputum [5].

Our objective was to measure the efficiency and accuracy of hybrid capture as a tool for identification of both fixed and minority variants in *Mtb*. First, we conducted an artificial strain mixture experiment in which we serially diluted DNA from well-characterized *Mtb* isolates in human DNA to simulate the overwhelmingly human DNA that is generated from clinical specimens. Second, we collected paired culture and sputum diagnostic samples from a national laboratory and compared the variation identified in paired samples.

## Methods

### Artificial mixture experiment using *Mtb* DNA standards

**Fig. 1.**
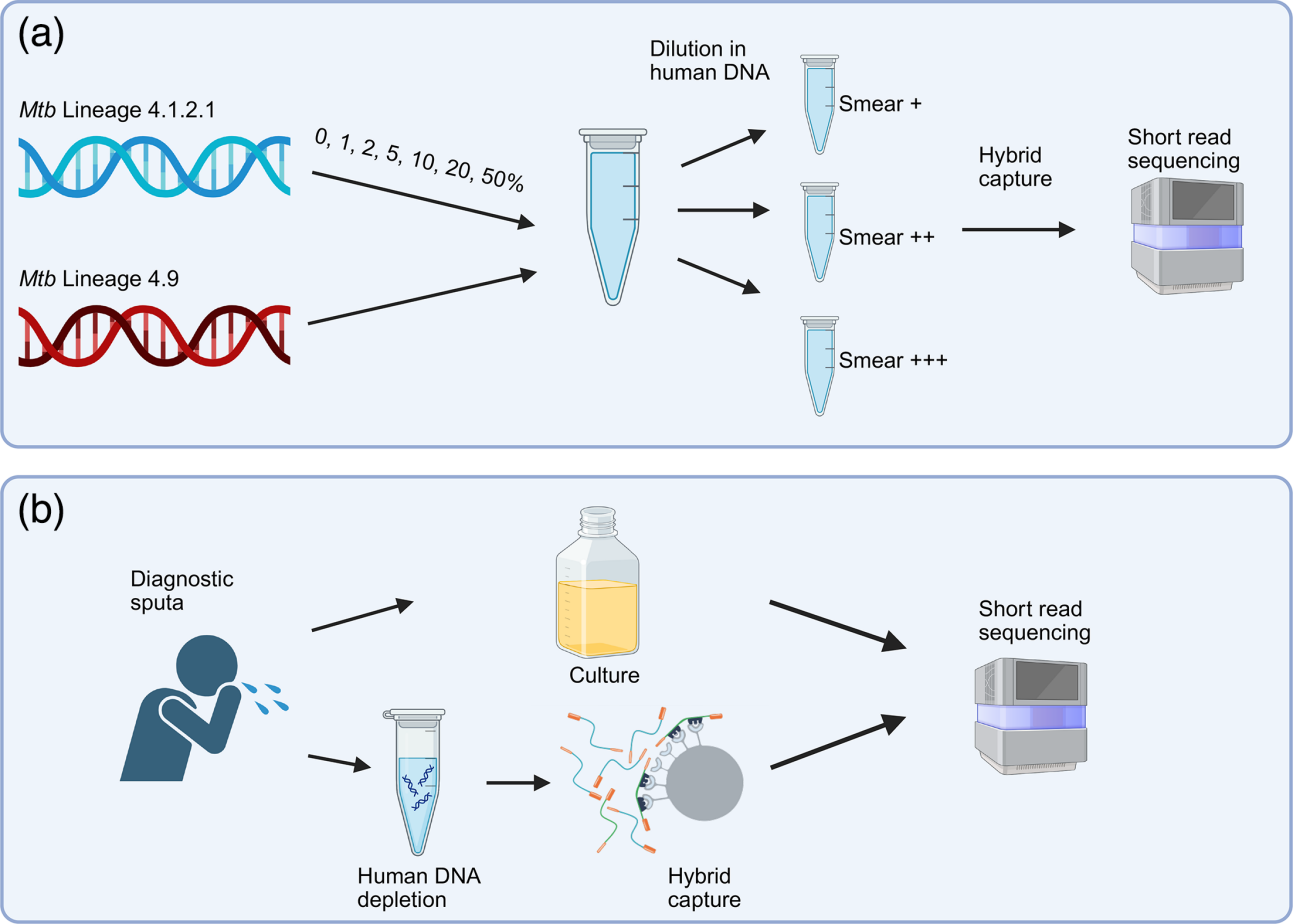
Experimental design. (**a**) We conducted a serial dilution experiment in which we mixed two *Mtb* DNA standards representing lineage 4.1.2.1 (strain X003899) and 4.9 (strain H37Ra), such that the minority strain represented 0–100% of the total DNA and then diluted *Mtb* DNA in human DNA to simulate clinical samples, which are overwhelmingly human DNA. We approximated the proportion of *Mtb* DNA found in sputum with smear microscopy grades 1+, 2+ and 3+ [7]. We used hybrid capture to enrich for *Mtb* DNA and conducted whole-genome sequencing of captured libraries on an Illumina platform. (**b**) We additionally prospectively sampled matched *Mtb* diagnostic cultures and unprocessed sputum submitted to ARUP diagnostic laboratories. We performed human DNA depletion, hybrid capture and Illumina whole-genome sequencing on matched samples.

To measure the efficiency of hybrid capture and accuracy of variant identification in captured samples, we created mixtures of DNA from reference strains H37Ra (ATCC 25177D-5) and X003899 (ATCC BAA-2237D-2) with well-characterized genomes (representing *Mtb* lineages 4.9 and 4.1.2.1), in which the minor strain comprised 0, 1, 2, 5, 10, 20 and 50% of the input DNA (Fig. 1). We selected H37Ra and X003899 because they were well-characterized *Mtb* DNA isolates, both of which were genetically distinct from the commonly used reference genome, H37Rv. Because both are lineage 4, they allow us to evaluate our ability to distinguish minority variants in closely related strains.

Mixed *Mtb* DNA was then diluted with a human DNA standard (HTB-22D, ATCC HTB-22) to simulate the high proportion of host DNA present in clinical sputum specimens. Dilutions were prepared on a mass basis using quantified DNA concentrations such that *Mtb* DNA comprised 0.01%, 0.03% or 0.3% of the total DNA input. These dilution levels were selected to approximate the range of *Mtb* DNA abundances observed in a shotgun metagenomic sequencing study of sputum specimens [7]. Specifically, estimates derived from Doughty *et al*. suggested mean *Mtb* DNA abundances of ~0.01%, 0.02% and 0.25% for smear grades 1+, 2+ and 3+, respectively [7]. Because these estimates were derived from a limited number of samples, we used approximate dilution levels spanning this range rather than attempting to reproduce the reported values exactly. For comparison, we also included a control dilution consisting of 100% *Mtb* DNA without human DNA. For each mixture and dilution level, we included a single replicate.

### Prospective sampling of paired sputum and cultured diagnostic samples

We additionally performed prospective genomic surveillance of paired sputa and diagnostic cultures at ARUP Laboratories, a national diagnostic laboratory in the USA (Fig. 1). Sputum samples were decontaminated. The sputum was used for standard mycobacteriology diagnostic culture and smear. Liquid cultures were performed using the Mycobacterial Growth Indicator Tube system. Positive MGIT cultures were identified as MTB complex using MALDI-TOF MS or 16S rRNA Sanger sequencing.

### Host DNA depletion and DNA extraction from sputum

An aliquot of decontaminated sputum was frozen at −80 °C until the time of processing. We followed a protocol from Kok *et al*. to deplete human DNA from sputum [9]. Prior to DNA extraction, 300 µl of decontaminated sputum samples were treated with 200 µl of a 2.5% saponin solution (Merck; diluted in 1× PBS and sterile filtered). Samples were vortexed and incubated at room temperature for 10 min to promote host cell lysis. Three hundred fifty microlitres of nuclease-free water (Merck) was added for 30 s, after which 12 µl of 5 M NaCl (Merck) was added to the samples to create an osmotic shock. Samples were centrifuged at 6,000 ***g*** for 5 min, and the pellets resuspended in 100 µl 1× PBS (Thermo Scientific).

Fifteen microlitres of DNase I [20,000 units (50 to 375 U µl−1), Thermo Scientific] and 11 µl of 10× DNase buffer (Thermo Scientific) were added to digest free DNA. Samples were incubated in a thermomixer (Eppendorf, Hamburg, Germany) with shaking at 800 r.p.m. at 37 °C for 30 min. After incubation, samples were centrifuged at 6,000 ***g*** for 3 min, and the pellet was washed twice with 800 µl 1× PBS [9, 21]. Finally, the pellet was resuspended in 300 µl of the lysis buffer of the extraction kit (Promega Maxwell RSC 48 Blood DNA kit) and extracted according to the manufacturer’s protocol [22].

We aliquoted 30 µl of DNA (target of ~250 ng DNA required for sequencing). We did not conduct mechanical lysis as part of DNA extraction.

### DNA extraction for cultured isolates

For the paired diagnostic culture samples, prior to DNA extraction, 75 µl of the culture suspension was treated with 15 µl Proteinase K solution and incubated at room temperature for 15 min. Seventy-five microlitres of lysis buffer was added and incubated at 95 °C for 30 min to kill viable mycobacterial cells. DNA from lysed samples was again extracted according to the manufacturer’s protocol [22].

### Phenotypic resistance profiles for clinical isolates

All baseline diagnostic isolates were phenotypically sensitive to isoniazid, rifampicin, ethambutol and pyrazinamide. Isolates were residual diagnostic samples submitted to ARUP and were deidentified for study purposes. Additional clinical information, including previous TB treatment history, is unavailable.

### Genomic sequencing and variant identification

We prepared genomic libraries with the NEB Ultra II FS DNA Prep with myBaits Enrichment TB (Arbor Biosciences, D10008Myctb1). We conducted hybrid capture of *Mtb* using a panel of biotinylated RNA baits tiled over the inferred *Mtb* ancestral genome [23], first published by Goig *et al*. [8] and which they have made available by request. We [23] followed the myBaits User Manual, version 5.02 [24] and pooled eight libraries, 250 ng DNA each, for a total of 2 µg per capture reaction, as recommended for small genome capture [24]. For clinical samples, we pooled seven libraries, 250 ng DNA each. We did not quantify Mtb genome copy number prior to pooling. We conducted paired-end sequencing on an Illumina NovaSeq X at the University of Utah High-Throughput Sequencing Core.

We identified SNP variants with the *mtb-call2* pipeline, as previously described [25]. Briefly, the pipeline taxonomically filters reads with Kraken [26], maps reads with *bwa* [27] and identifies genomic variants with *GATK* [28]. *GATK HaplotypeCaller* excludes secondary alignments by default [28].

To minimize artefacts arising from ambiguous read mappings that have been previously described in *Mtb* [5], supplementary alignments were excluded from BAM files prior to variant identification. Supplementary alignments occur when a single sequencing read is split into two or more segments and aligned and may reflect underlying structural variation as well as errors. We evaluated the impact of supplementary alignment filtering by comparing minority variant calls obtained from analyses performed with and without supplementary alignments (Fig. S1, available in the online Supplementary Material). Because exclusion of supplementary alignments reduced the number of minority variants detected, particularly in capture-derived samples, all subsequent analyses were conducted using primary alignments only.

Many *Mtb* genomic analysis pipelines routinely exclude the repetitive genes from the proline-glutamic acid and proline-proline-glutamic acid families, known as PE/PPE genes, as they are difficult for mapping algorithms and error-prone. We, therefore, stratified analysis by genomic regions outside and within the PE/PPE genes. We additionally excluded the ribosomal RNA genes (*rrs* and *rrl*), which are highly conserved across bacterial species and previously reported to contribute to ambiguous mapping [11]. Genome-wide coverage metrics were assessed using Picard WgsMetrics. Coverage uniformity was evaluated using the FOLD_80_BASE_PENALTY metric and the proportion of genomic bases achieving predefined coverage thresholds (Fig. S2) [29].

### Statistical analysis

Sequencing known mixtures of two *Mtb* DNA standards allowed us to use existing genomic resources to generate a ‘truth’ variant call format (VCF) file of variants in H37Ra and X003899 with respect to the reference genome H37Rv. To do this, we used whole-genome sequences from H37Ra (GCF_001938725.1) and X003899 (supplied by ATCC). We used *MUMmer* to pairwise align each genome with the H37Rv genome and identified SNP variants using *nucmer* [30]. We measured the performance (precision, recall and F1 score) of variants identified in the serial dilution experiment using *hap.py*, a performance benchmarking tool for variant identification [31]. We assessed the correlation between the expected minor allele frequency (proportion minority strain in the input mixture) and observed minor allele frequency for minority variants with Pearson’s correlation coefficient. We assigned true and false positive variants based on the majority allele in the input mixture.

For the artificial mixture samples and the diagnostic samples, we determined the percentage of reads taxonomically classified to the *Mycobacterium* genus by Kraken [26], the percentage of reads mapping to *Mtb* after filtering and mean coverage with *Picard* CollectWgsMetrics [29]. We additionally identified taxa outside of *Mycobacterium* with a large proportion of assigned sequence reads.

For the genomic surveillance samples, we quantified the number of minority variants as biallelic variants with a depth of 10× or greater for reference and alternative alleles. We measured the concordance of SNPs identified in cultured and captured samples with Jaccard’s index and measured the association between minor allele frequency for minority variants identified in both paired cultured and captured samples with Pearson’s correlation coefficient.

## Results

**Fig. 2.**
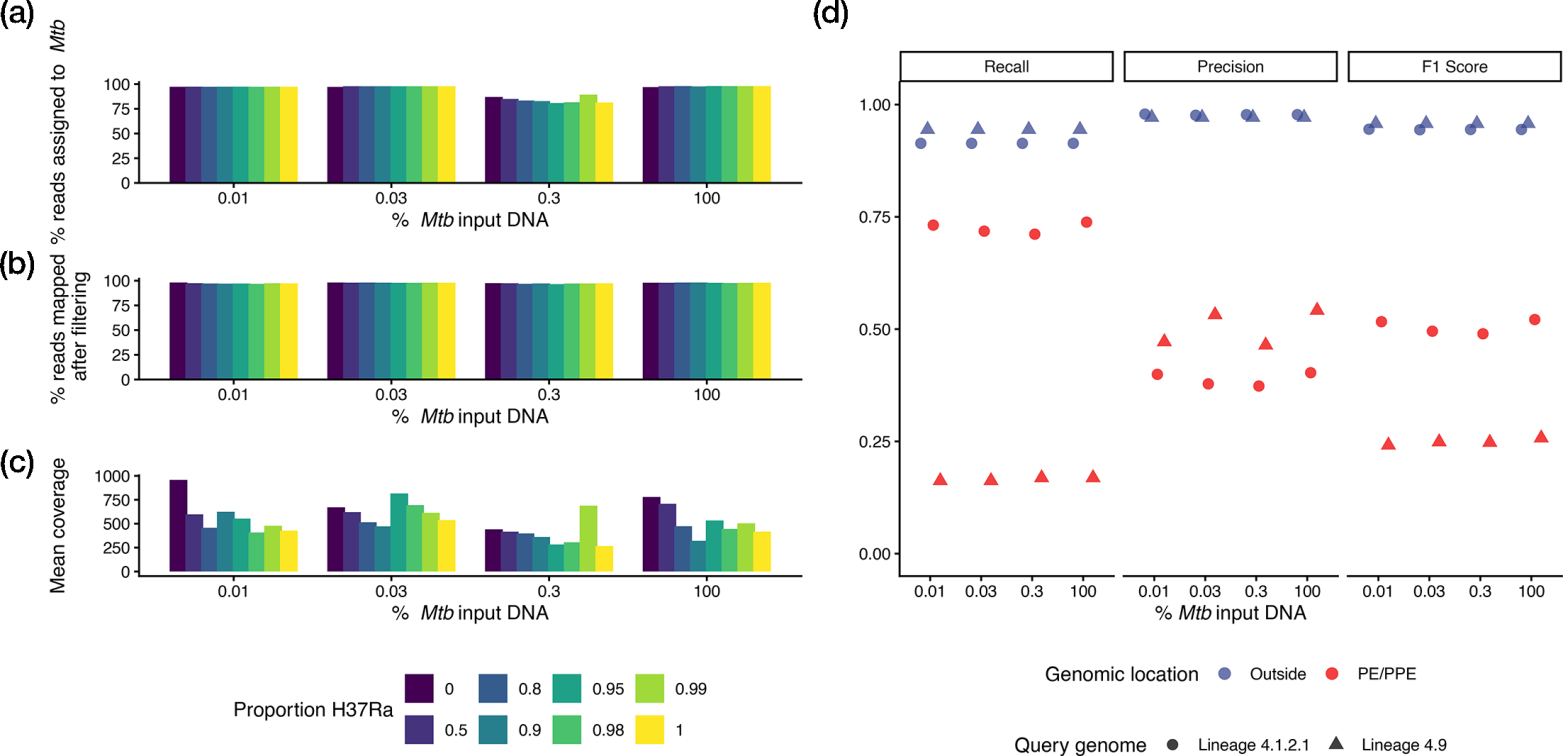
Performance of hybrid capture for experimental strain mixtures. (**a**) The percentage of reads taxonomically classified to the *Mycobacterium* genus by Kraken, (**b**) the percentage of reads mapping to *Mtb* after filtering and mean coverage. The *x*-axis for both (a, b and c) indicates the percentage of input *Mtb* DNA in the total DNA mixture, and colour indicates the proportion of H37Ra (colour). (**d**) Precision and recall (sensitivity) of SNP identification, by percentage of *Mtb*, for the samples comprised of 100% lineage 4.1.2.1 (points) and 100% lineage 4.9 (triangles). Colours indicate genomic region: outside PE/PPE genes, blue; within PE/PPE genes, red.

In the artificial strain mixture experiment, taxonomic assignment of reads to the *Mycobacterium* genus was high, even when *Mtb* comprised a small proportion of the input DNA (97% of reads were assigned to *Mycobacterium*, when *Mtb* DNA comprised 0.01% of the input) (Fig. 2a). The remaining assigned reads were largely human derived [mean, 0.3%; interquartile range (IQR), 2.6%] (Fig. S3). While the majority of samples have very little human-derived DNA (<1%), the samples comprising 0.3% *Mtb* DNA have higher levels of human DNA, possibly reflecting a pipetting error (Fig. S2). After taxonomic filtering, an average of 98% of *Mtb* reads mapped to the H37Rv reference genome (Fig. 2b). Taxonomic assignment and read-mapping efficiency after taxonomic filtering varied with the proportion of *Mtb* in the input sample as well as the genomic background (Fig. 2). Mean genomic coverage was high (mean, 525; IQR, 205) across all samples (Fig. 2c).

**Fig. 3.**
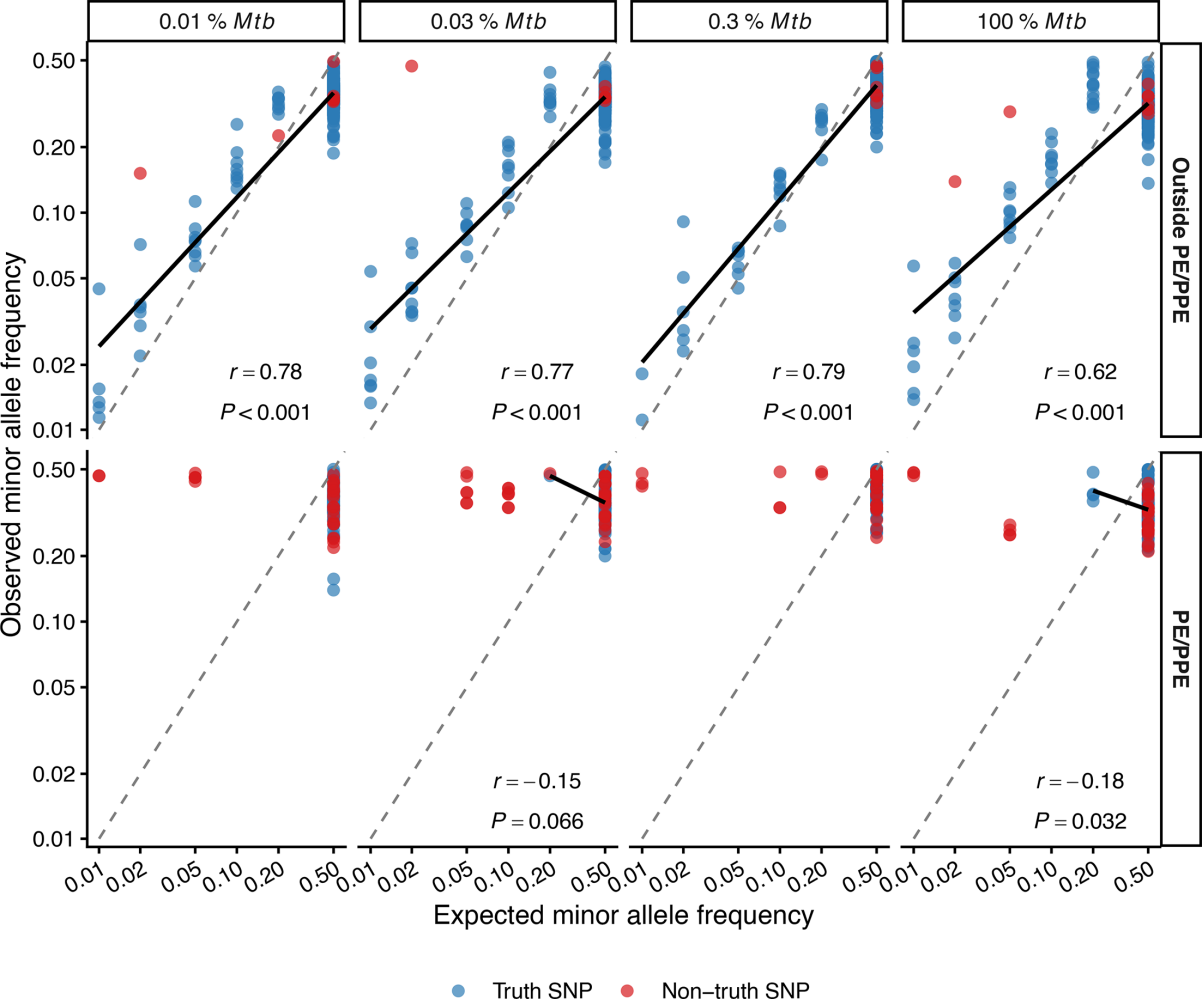
Proportion of minor strain (expected minor allele frequency) vs. observed minor allele frequency for minority variants identified, with columns indicating the percentage of *Mtb* DNA in the input DNA before hybrid capture and rows indicating genomic location. Colours indicate minor allele status.

SNP identification via hybrid capture had a mean recall of 0.91 and mean precision of 0.98 for the lineage 4.1.2.1 standard and a recall of 0.94 and precision of 0.97 for the lineage 4.9 standard, outside of PE/PPE genes (Fig. 2d). In PE/PPE genes, mean recall was 0.72 and precision of 0.39 for the lineage 4.1.2.1 standard, and recall was 0.17 and precision was 0.50 for the lineage 4.9 standard. Neither recall (*P*=0.80) nor precision (*P*=0.43) was impacted by the percentage of *Mtb* DNA in the input sample in separate binomial models. Observed minor allele frequencies were closely correlated with expected minor allele frequencies (proportions of the minor strain) across dilutions (*r*=0.62–0.79, *P*<0.001) outside of PE/PPE genes (Fig. 3).

**Fig. 4.**
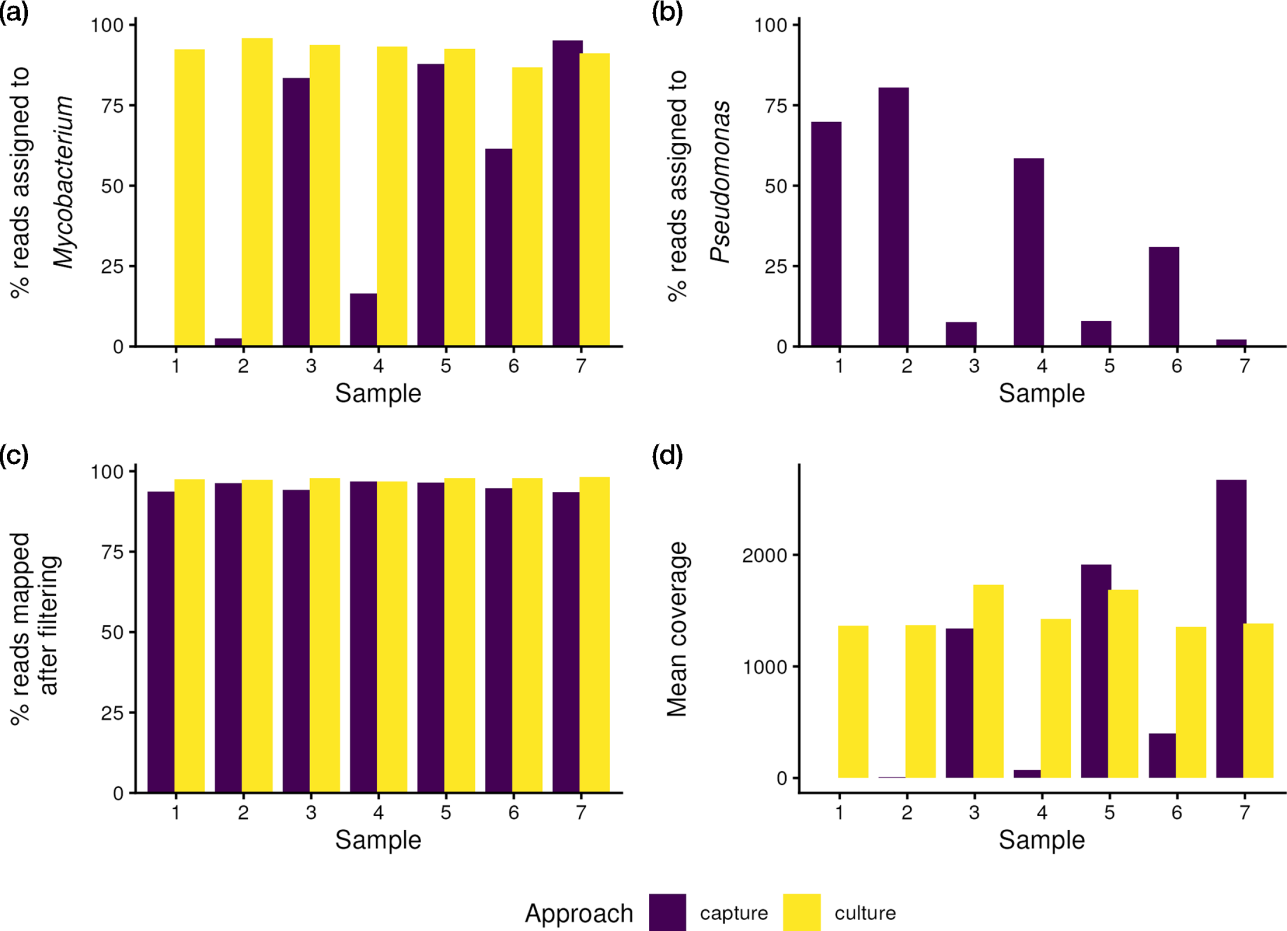
Performance of hybrid capture and culture for diagnostic sputum samples. For diagnostic sputum samples, (**a**) the percentage of reads taxonomically classified to the *Mycobacterium* genus by Kraken for paired hybrid-captured and cultured samples, (**b**) the percentage of reads taxonomically classified to the *Pseudomonas* genus by Kraken, (**c**) the percentage of reads mapping to *Mtb* after filtering and (d) mean coverage of the *Mtb* genome. Colour indicates approach for *Mtb* enrichment: hybrid capture and culture.

To then evaluate hybrid capture on clinical samples, we also compared clinical diagnostic cultures with matched hybrid-captured samples. Clinical samples were genomically diverse, including representatives from four global *Mtb* lineages [with highest resolution sublineage assignments of 1.2.1.2; 2.2.1; 2.2.1.1 (2 isolates); 3; 4.1.1.3; and 4.7]. Among clinical specimens, cultured samples had a higher percentage of reads assigned to *Mtb* (mean, 92%) than did paired captured samples (mean, 48%), prior to filtering. Four of seven captured samples had less than 75% of reads assigned to *Mtb* (Fig. 4a). We investigated taxonomic assignments for all captured samples and found that the majority of non-*Mtb* reads were assigned to the genus *Pseudomonas*, and largely, *Pseudomonas fluorescens*, a common environmental bacterium (Fig. 4b). After taxonomic filtering, the majority of reads for all captured samples mapped to *Mtb*, though mapping efficiency remained higher for cultured (98% reads) compared to captured samples (95% reads). Two samples had extremely low, <10× coverage of the *Mtb* genome. Contamination levels were inversely correlated with coverage: mean coverage of the four samples that had >25% *Pseudomonas* contamination was 120×, compared to the mean coverage of 1,980× for other samples, comparable to the depth of the cultured samples (1,470×) (Fig. 4d). Human-derived DNA makes up a very low proportion of all samples (<0.5%, Fig. S3).

**Fig. 5.**
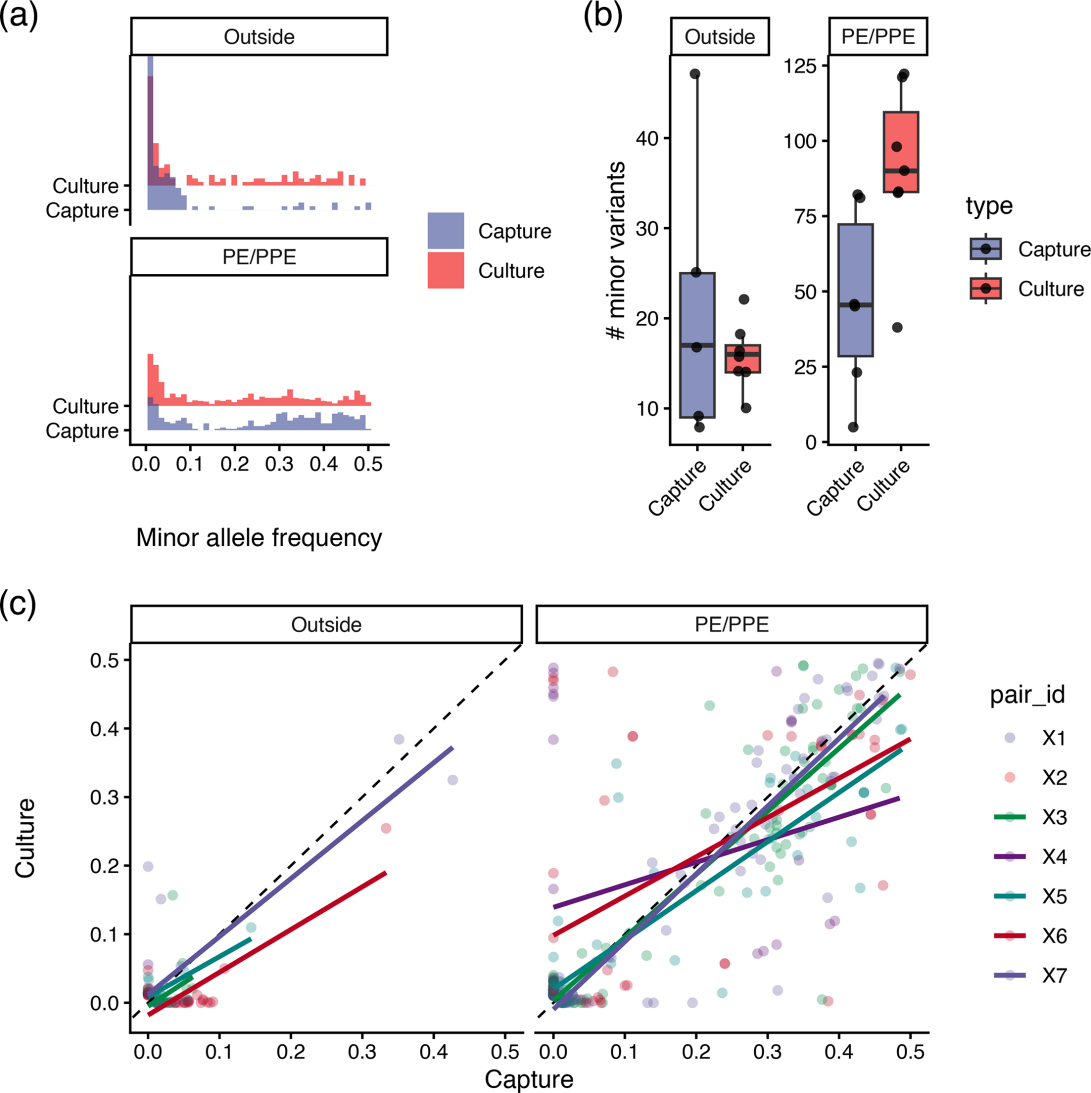
Minority variation recovered in paired clinical *Mtb* isolates. Below, we include all samples, regardless of *Pseudomonas* contamination. (**a**) Minor allele frequency distribution for cultured and captured samples. (**b**) The number of minor variants. Points indicate sample variant counts and box plots indicate the median, interquartile range and whiskers extend to points within 1.5 times the IQR from the edges of the box. For visualization, the *y*-axis is on a log scale. (**c**) Correlation of minor allele frequency between paired cultured and captured samples, with best fit lines drawn for each sample. Pearson’s correlation coefficient and *P*-value are reported by genomic location. No within-host variants were identified outside PE and PPE genes for sample X3.

We identified minority variants in each genomic surveillance sample with a minor allele frequency of at least 1% and minor allele depth of coverage of at least 5× (Fig. 5a). Few minority variants were consistently identified between cultured and captured samples, ranging from 0% (0 variants identified by both cultured and captured sample out of a union of 30 unique variants identified) to 15% (3/20 variants) outside PE and PPE genes (mean, 7%). Minority variants were more consistently identified by culture and capture in PE and PPE genes, ranging from 0% (0/83) to 41% (48/116) shared (mean, 18%). We did not find a difference between the number of minority variants detected in cultured (median, 16 variants; range, 10–22 variants) and captured samples (median, 17 variants; range, 8–47 variants) (paired Wilcoxon test, *P*=1) outside of PE/PPE genes (Fig. 5b). Within PE/PPE genes, more minority variants were identified through culture (median, 90; range, 38–122) than hybrid-captured samples (median, 46; range, 5–82; *P*=0.03). Minor allele frequencies of minor variants identified in both culture and captured samples were correlated both outside (*r*=0.87, *P*≤0.001) and within the PE/PPE genes (*r*=0.71, *P*<0.001) (Fig. 5c).

Our findings were largely consistent when we excluded the four samples with a high degree of *Pseudomonas* contamination (>25% *Pseudomonas*) (Fig. S4). Few minority variants were consistently identified between cultured and captured samples, ranging from 8% (3 variants identified by both cultured and captured sample out of a total of 20 unique variants identified) to 15% (4/53 variants) outside PE/PPE genes (mean, 12% shared). Minority variants were more consistently identified by culture and capture in PE/PPE genes, ranging from 27% (31/113) to 41% (48/126) shared (mean, 35%). We did not find a difference between the number of minority variants detected in cultured (median, 14 variants; range, 10–18 variants) and captured samples (median, 17 variants; range, 9–47 variants) (paired Wilcoxon test, *P*=1) outside of PE/PPE genes, nor within PE/PPE genes (*P*=0.25) (Fig. S4). Minor allele frequencies of minor variants identified in both culture and captured samples were correlated both outside PE/PPE (*r*=0.87, *P*<0.001) and within the PE/PPE genes (*r*=0.79, *P*<0.001) (Fig. S4).

## Discussion

Genomic epidemiology is a powerful tool for understanding *Mtb* transmission dynamics and rates of antibiotic resistance but largely relies on mycobacterial culture, which is time-consuming, requires specialized laboratory facilities and may introduce a selective filter on the *Mtb* population. We evaluated hybrid capture as a potential replacement for culture. We found that hybrid capture enables efficient and accurate variant identification, even in samples overwhelmed by human DNA, and that it can be used to recover minority variation. Our findings indicate that hybrid capture is a promising tool for *Mtb* population genomic sequencing directly from diagnostic sputum samples that could expand the coverage of *Mtb* genomic epidemiology.

Previous studies have reported that hybrid capture can be used to sequence high-coverage *Mtb* genomes directly from clinical specimens, generate genotypic resistance predictions concordant with phenotypic resistance and support transmission analyses, possibly more quickly than culture-based sequencing [5, 8, 8, 13, 14]. Other studies have reported the recovery of minor variation in hybrid-captured samples, but the effect of hybrid capture on the magnitude of recovered minority variation has been inconclusive. One study reported greater levels of within-host variation identified in captured samples compared to matched cultures [15], while another reported that minor variation identified by capture largely reflects what is present in culture [8]. However, the efficiency of hybrid capture has not been systematically evaluated across serial dilutions of *Mtb* DNA in a human DNA background, nor has its performance in identifying fixed and minority variants been benchmarked using defined mixtures of well-characterized isolates.

Our findings have important implications for genomic epidemiology studies relying on hybrid capture. Previous studies have demonstrated that hybrid capture use can enable whole-genome sequencing even in samples with low bacterial burden, including smear-negative sputa [8, 15]. Consistent with these studies, we found that hybrid capture can be used for efficient sequencing, even when *Mtb* DNA is present at levels that reflect low bacterial burden or smear grade 1+. As expected, variant identification was more accurate for the lineage 4.9 isolate than the lineage 4.1.2.1 isolate, reflecting that capture probes were designed with the *H37Rv* reference genome, also in lineage 4.9. This highlights a key bias in hybrid capture that probes are based on previously characterized *Mtb* DNA and, therefore, may not be able to hybridize with genomic insertions relative to the reference used in probe design. We previously found that the accuracy of *Mtb* variant identification decreased with increasing distance between the query and reference genome [28]. We expect that hybrid capture efficiency may similarly decrease with increasing distance between the query and reference genome, reflecting decreasing hybridization with greater genetic distance. However, panels that tile lineage-defining positions and drug-resistance regions more densely [11] might mitigate this reference bias [11].

As expected, both recall and precision dropped in PE and PPE genes, known to be difficult to map and often excluded from *Mtb* genomic analysis [32]. The higher recall of lineage 4.1.2.1 compared to lineage 4.9 variation in PE and PPE genes likely reflects the lower magnitude of true positive variation detected in pairwise alignment.

We find that bacterial contamination can overwhelm *Mtb* sequencing reads, particularly in samples with low *Mtb* DNA input. In these cases, low copy number likely limits capture efficiency and increases susceptibility to off-target enrichment from background organisms. The prominence of *Pseudomonas* reads in a subset of samples suggests competitive interference during capture, reducing recovery of *Mtb* sequences. Although the source of *Pseudomonas* is unclear, it may have been introduced during sample processing or hybrid capture, as all samples were handled in the same laboratories. Notably, this effect was cohort-specific and not observed across all datasets, suggesting it is not a general limitation of the approach.

Taxonomic filtering is an important step both to exclude sequence reads that do not map to the *Mycobacterium* genus as well as to identify samples with high levels of contamination that may need to be excluded from analysis [33]. Yet, taxonomic filtering is imperfect. In contaminated samples, we observed minority variation that persisted after filtering was concentrated in the *rrs* or 16S gene, which is highly conserved between *Mtb* and *Pseudomonas*. Excluding conserved microbial genes like *rrs* from variant identification in hybrid capture may be important. Further, even among samples with high levels of contamination, mapping efficiency, or the percentage of reads mapping to *Mtb* after taxonomic filtering, was high. Mapping efficiency thus is not a useful metric for identifying contamination, and sample-level filters that exclude samples with high levels of contamination are needed. For example, we detected high levels of minority variation in samples with high levels of *Pseudomonas* contamination, likely reflecting errors associated with incomplete taxonomic filtering of reads. However, our findings did not substantially change when excluding highly contaminated samples.

Our findings also support the use of hybrid capture for minor variant detection. In our serial dilution experiment, we found that input and observed minor allele frequencies were closely correlated in the serial dilution samples. Similarly, minor allele frequencies were correlated between hybrid capture and cultured diagnostic sputum samples, evidence that hybrid capture can effectively capture two alleles at a genomic locus. Input and observed minor allele frequencies between captured and cultured samples are also correlated within the PE and PPE genes. Although false-positive errors are also concentrated here, this highlights that excluding such variation likely excludes important biologically and epidemiologically meaningful signal [28]. We observed high variability in the number of minor variants recovered in captured clinical samples as well as low levels of concordance between minor variants in capture and cultured samples. This could reflect true biological differences between the sample types, such as the previously described loss of variation in culture [2], and variation in within-host diversity between samples, or reflect biases in either approach, such as the impact of contaminating reads on variant detection. However, the underlying true pattern of variation for diagnostic samples is unknown, and, therefore, we cannot measure the accuracy of minority variant identification [28].

Our study has several limitations. First, we included only a small sample size of paired cultured and captured samples that were not contaminated. Studies with larger sample sizes are needed to establish the concordance of minority variants identified via culture and capture and to identify specific variant filters or mapping approaches that enable the recovery of high-quality minority variants from hybrid-captured samples. Second, one of the limitations of hybrid capture and amplicon sequencing is that these approaches capture variation that is closely related to the reference genome used for probe or primer design. Such approaches may not be sensitive to large insertions or genomic rearrangements in samples with respect to the reference genome. Approaches that focus on host DNA depletion and metagenomic sequencing may be more effective in these cases. Third, probe synthesis for hybrid capture remains expensive, although some groups have reported decreasing per-sample costs [8]. Optimizing cheaper alternatives and making *Mtb* probe design publicly available will increase the possibility that it can become a routine tool in population genomic sequencing and a viable tool for public health. Fourth, artificial mixtures of human and *Mtb* DNA do not fully reproduce the complexity of clinical sputum samples, which also contain diverse microbial DNA and may overestimate capture efficiency. We include clinical samples to address this.

Several different factors, including *Mtb* lineage, the sample microbiota and sample pre-processing steps [11, 34], all influence hybrid capture efficiency as well as the accuracy of variant identification. Further studies are needed to optimize cost-effective and scalable protocols to generate *Mtb* genomes directly from clinical samples.

In conclusion, we find that hybrid capture efficiently enriches *Mtb* DNA from samples that are overwhelmingly human DNA and can accurately identify both consensus and minor variants. Hybrid capture offers an alternative to traditional culture-based sequencing workflows, enabling rapid genomic analysis without the need for mycobacterial culture facilities.

## Supplementary material

10.1099/mgen.0.001806 Supplementary Material 1.

## Abbreviations

IQR: interquartile range
*Mtb*: *Mycobacterium tuberculosis*
