# Supplementary material for: Optimizing culture-free approaches to recover high-quality Mycobacterium tuberculosis genomic variation
Source: Microb Genom. 2026 Aug 18;12(8):001806. doi: 10.1099/mgen.0.001806 (PMC13485273; doi:10.1099/mgen.0.001806)
Supplement: Supplementary file 1 [file mgen-12-01806-s001.pdf]

Supplementary Information

Supplementary Figure 1. Impact of supplementary alignment filtering on minority variant identification. Box plots indicate the change in number of minority variants in BAM files in which supplementary alignments were filtered as compared to full BAM files. Points indicate sample variant counts and box plots indicate the median, interquartile range, and whiskers extend to points within 1.5 times the IQR from the edges of the box. Facets indicate genomic strata and color indicates capture versus cultured samples. Excluding supplementary alignments as previously described[1] reduced the number of minority variants detected, with the largest effect observed among capture samples and variants outside PE/PPE loci. Variants within *rrs/rrl* were largely unaffected. In the main analysis, we exclude supplementary alignments.

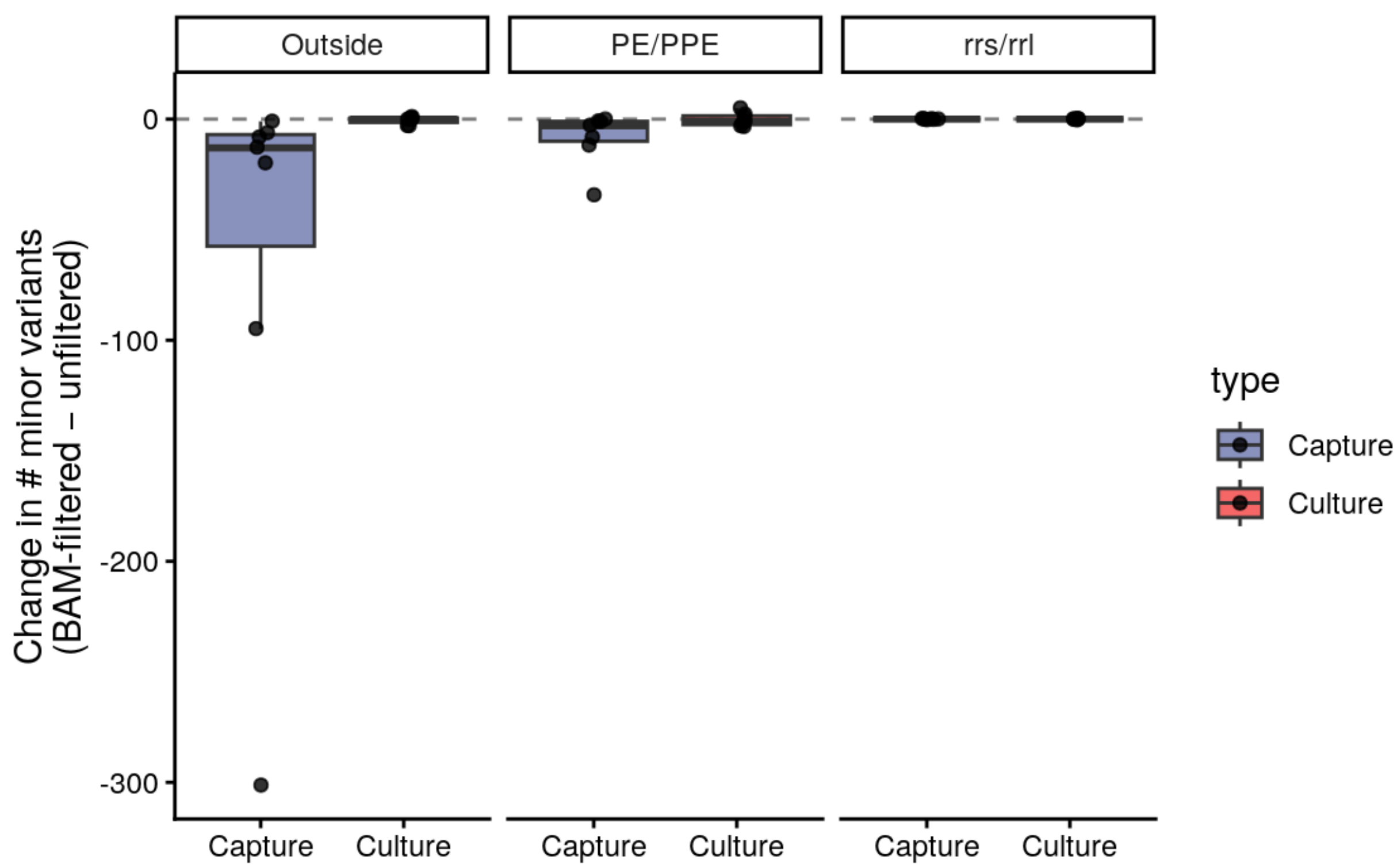

Supplementary Figure 2. Sample mean coverage and coverage heterogeneity by approach. Points indicate per-sample values and box plots indicate the median, interquartile range, and whiskers extend to points within 1.5 times the IQR from the edges of the box. Coverage heterogeneity is measured by the Picard Tools coverage metric "FOLD_80_BASE_PENALTY," which indicates the degree of additional sequencing that would be needed so that 80% of target bases reach the mean coverage. Colors indicate approach. The y-axis in (b) is cut off at 5, excluding one outlier point. Coverage was assessed along the full genome. Coverage was broadly uniform across samples, with coverage heterogeneity as measured by median FOLD_80_BASE_PENALTY values of approximately 2.4 for hybrid capture samples, 1.1 for cultured samples, and 2.7 for serial dilution samples, indicating only modest genome-wide coverage heterogeneity. Across all sample groups, a median of 99% of the genome was covered at ≥10X coverage.

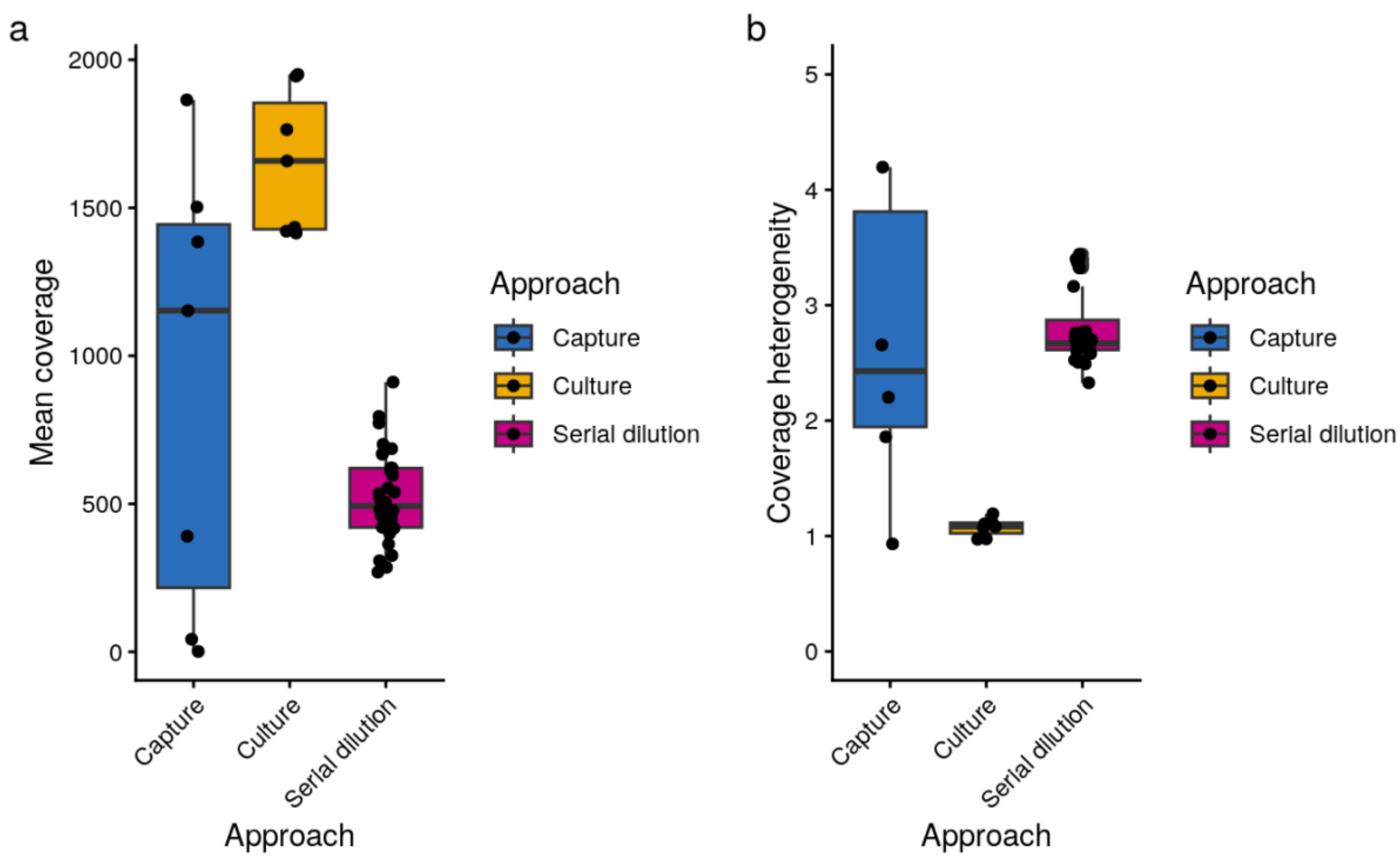

Supplementary Figure 3. Human-derived reads across approaches. The percentage of reads assigned to *Homo sapiens* for (a) the artificial mixture experiment, and (b) clinical isolates. The x-axis for (a) indicates the percentage input *Mtb* DNA in the total DNA mixture and color indicates the proportion H37Ra (color). For (b), color indicates approach for *Mtb* enrichment: hybrid capture and culture.

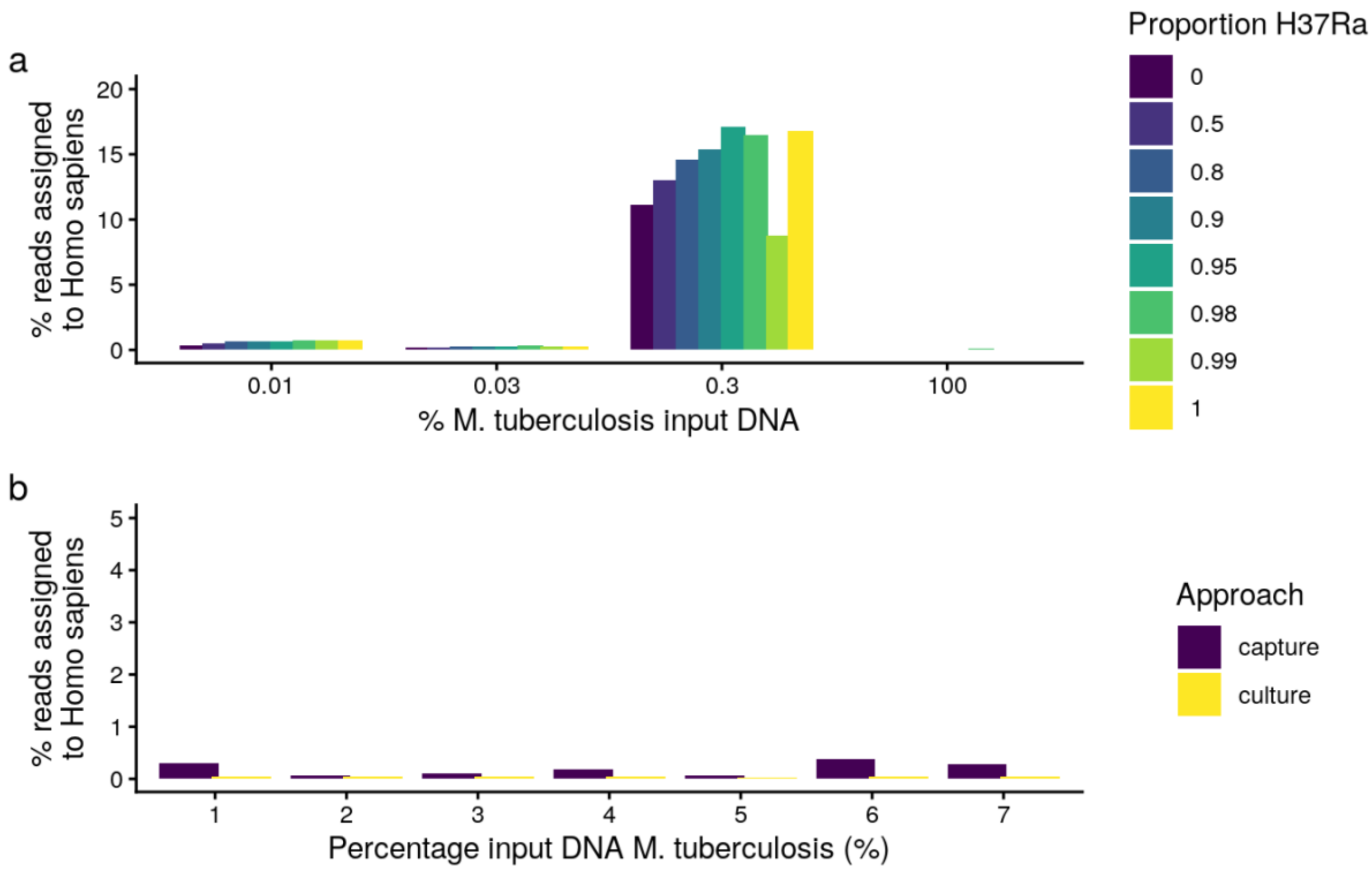

Supplementary Figure 4. Minor variants recovered in paired clinical *Mtb* isolates. We include variants with a minor allele frequency of 1% and with minor allele support of at least 5X. Below, we include the three paired samples with less than 25% *Pseudomonas* contamination; Figure 5 includes all samples. (a) Minor allele frequency distribution for cultured and captured samples. (b) The number of minor variants. Points indicate sample variant counts and box plots indicate the median, interquartile range, and whiskers extend to points within 1.5 times the IQR from the edges of the box. (c) Correlation of minor allele frequency between paired cultured and captured samples, with best fit lines drawn for each sample. Pearson's correlation coefficient and p-value are reported by genomic location. No minor variants were identified outside PE and PPE genes for sample X3.

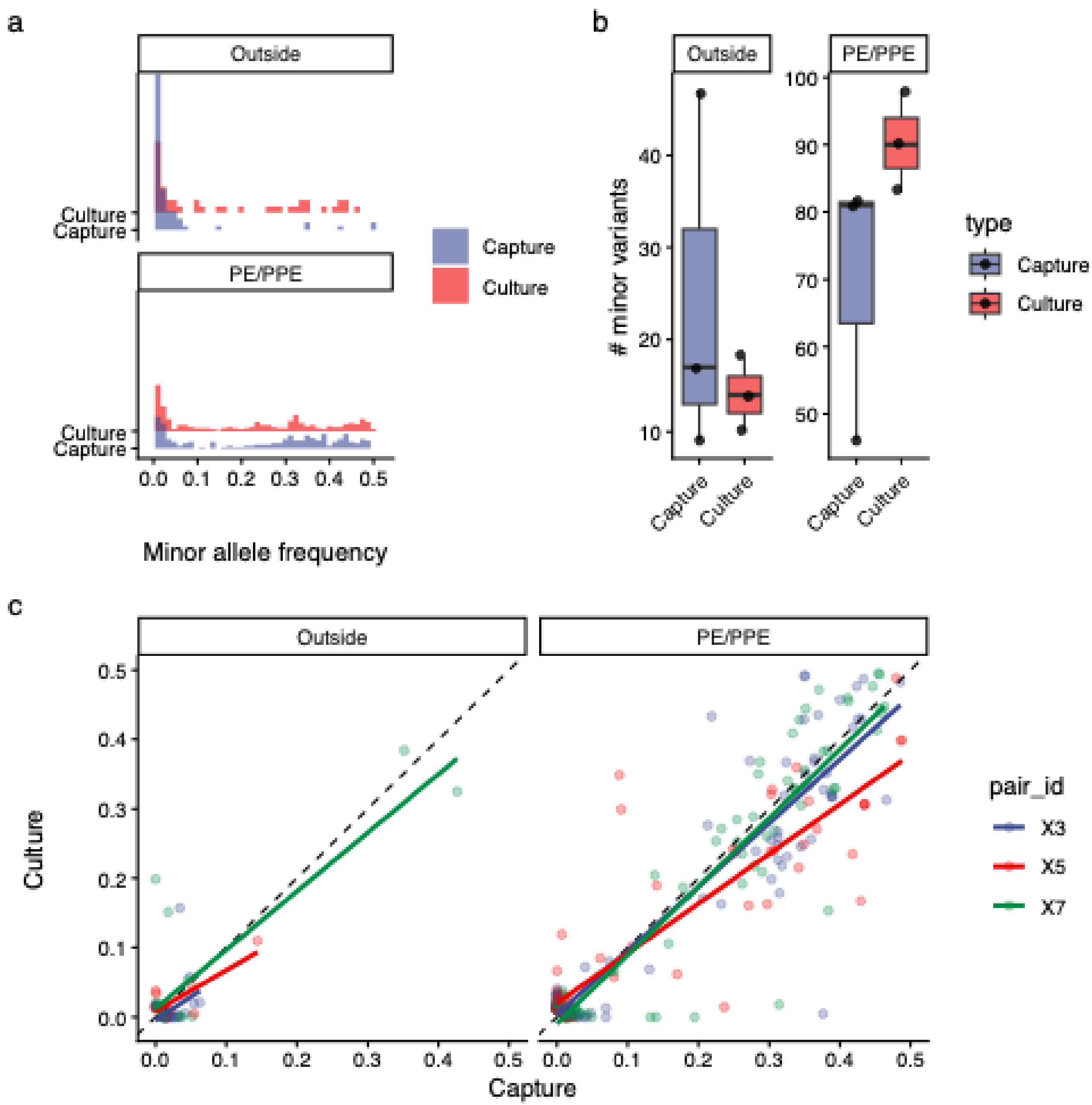

Supplementary Table 1. Sample information. Short read sequence archive (SRA) accession numbers for genomic data, sample names, SPUID, organism name, taxonomic ID, and isolate information for all samples. Sample names beginning with 22623 are those in the artificial strain mixture experiment, 22603 are cultured diagnostic samples, and 22617 are hybrid captured diagnostic samples.

Accession	Sample Name	SPUID	Organism	Tax ID	Isolate
SAMN54205463	22623X1	22623X1	Mycobacterium tuberculosis	1773	100% H37Ra DNA (ATCC) mixed with 0% X003899 DNA (ATCC) .
SAMN54205464	22623X2	22623X2	Mycobacterium tuberculosis	1773	99% H37Ra DNA (ATCC) mixed with 1% X003899 DNA (ATCC) .
SAMN54205465	22623X3	22623X3	Mycobacterium tuberculosis	1773	98% H37Ra DNA (ATCC) mixed with 2% X003899 DNA (ATCC) .
SAMN54205466	22623X4	22623X4	Mycobacterium tuberculosis	1773	95% H37Ra DNA (ATCC) mixed with 5% X003899 DNA (ATCC) .
SAMN54205467	22623X5	22623X5	Mycobacterium tuberculosis	1773	90% H37Ra DNA (ATCC) mixed with 10% X003899 DNA (ATCC) .
SAMN54205468	22623X6	22623X6	Mycobacterium tuberculosis	1773	80% H37Ra DNA (ATCC) mixed with 20% X003899 DNA (ATCC) .
SAMN54205469	22623X7	22623X7	Mycobacterium tuberculosis	1773	50% H37Ra DNA (ATCC) mixed with 50% X003899 DNA (ATCC) .
SAMN54205470	22623X8	22623X8	Mycobacterium tuberculosis	1773	0% H37Ra DNA (ATCC) mixed with 100% X003899 DNA (ATCC) .
SAMN54205471	22623X9	22623X9	Mycobacterium tuberculosis	1773	100% H37Ra DNA (ATCC) mixed with 0% X003899 DNA (ATCC) .
SAMN54205472	22623X10	22623X10	Mycobacterium tuberculosis	1773	99% H37Ra DNA (ATCC) mixed with 1% X003899 DNA (ATCC) .
SAMN54205473	22623X11	22623X11	Mycobacterium tuberculosis	1773	98% H37Ra DNA (ATCC) mixed with 2% X003899 DNA (ATCC) .
SAMN54205474	22623X12	22623X12	Mycobacterium tuberculosis	1773	95% H37Ra DNA (ATCC) mixed with 5% X003899 DNA (ATCC) .
SAMN54205475	22623X13	22623X13	Mycobacterium tuberculosis	1773	90% H37Ra DNA (ATCC) mixed with 10% X003899 DNA (ATCC) .
SAMN54205476	22623X14	22623X14	Mycobacterium tuberculosis	1773	80% H37Ra DNA (ATCC) mixed with 20% X003899 DNA (ATCC) .
SAMN54205477	22623X15	22623X15	Mycobacterium tuberculosis	1773	50% H37Ra DNA (ATCC) mixed with 50% X003899 DNA (ATCC) .
SAMN54205478	22623X16	22623X16	Mycobacterium tuberculosis	1773	0% H37Ra DNA (ATCC) mixed with 100% X003899 DNA (ATCC) .
SAMN54205479	22623X17	22623X17	Mycobacterium tuberculosis	1773	100% H37Ra DNA (ATCC) mixed with 0% X003899 DNA (ATCC) .
SAMN54205480	22623X18	22623X18	Mycobacterium tuberculosis	1773	99% H37Ra DNA (ATCC) mixed with 1% X003899 DNA (ATCC) .
SAMN54205481	22623X19	22623X19	Mycobacterium tuberculosis	1773	98% H37Ra DNA (ATCC) mixed with 2% X003899 DNA (ATCC) .
SAMN54205482	22623X20	22623X20	Mycobacterium tuberculosis	1773	95% H37Ra DNA (ATCC) mixed with 5% X003899 DNA (ATCC) .
SAMN54205483	22623X21	22623X21	Mycobacterium tuberculosis	1773	90% H37Ra DNA (ATCC) mixed with 10% X003899 DNA (ATCC) .
SAMN54205484	22623X22	22623X22	Mycobacterium tuberculosis	1773	80% H37Ra DNA (ATCC) mixed with 20% X003899 DNA (ATCC) .
SAMN54205485	22623X23	22623X23	Mycobacterium tuberculosis	1773	50% H37Ra DNA (ATCC) mixed with 50% X003899 DNA (ATCC) .

SAMN54205486	22623X24	22623X24	Mycobacterium tuberculosis	1773	0% H37Ra DNA (ATCC) mixed with 100% X003899 DNA (ATCC) .
SAMN54205487	22623X25	22623X25	Mycobacterium tuberculosis	1773	100% H37Ra DNA (ATCC) mixed with 0% X003899 DNA (ATCC) .
SAMN54205488	22623X26	22623X26	Mycobacterium tuberculosis	1773	99% H37Ra DNA (ATCC) mixed with 1% X003899 DNA (ATCC) .
SAMN54205489	22623X27	22623X27	Mycobacterium tuberculosis	1773	98% H37Ra DNA (ATCC) mixed with 2% X003899 DNA (ATCC) .
SAMN54205490	22623X28	22623X28	Mycobacterium tuberculosis	1773	95% H37Ra DNA (ATCC) mixed with 5% X003899 DNA (ATCC) .
SAMN54205491	22623X29	22623X29	Mycobacterium tuberculosis	1773	90% H37Ra DNA (ATCC) mixed with 10% X003899 DNA (ATCC) .
SAMN54205492	22623X30	22623X30	Mycobacterium tuberculosis	1773	80% H37Ra DNA (ATCC) mixed with 20% X003899 DNA (ATCC) .
SAMN54205493	22623X31	22623X31	Mycobacterium tuberculosis	1773	50% H37Ra DNA (ATCC) mixed with 50% X003899 DNA (ATCC) .
SAMN54205494	22623X32	22623X32	Mycobacterium tuberculosis	1773	0% H37Ra DNA (ATCC) mixed with 100% X003899 DNA (ATCC) .
SAMN54205495	22603X1	22603X1	Mycobacterium tuberculosis	1773	1_Culture
SAMN54205496	22603X2	22603X2	Mycobacterium tuberculosis	1773	2_Culture
SAMN54205497	22603X3	22603X3	Mycobacterium tuberculosis	1773	3_Culture
SAMN54205498	22603X4	22603X4	Mycobacterium tuberculosis	1773	4_Culture
SAMN54205499	22603X5	22603X5	Mycobacterium tuberculosis	1773	5_Culture
SAMN54205500	22603X6	22603X6	Mycobacterium tuberculosis	1773	6_Culture
SAMN54205501	22603X7	22603X7	Mycobacterium tuberculosis	1773	7_Culture
SAMN54205502	22617X1	22617X1	Mycobacterium tuberculosis	1773	1_Sputum
SAMN54205503	22617X2	22617X2	Mycobacterium tuberculosis	1773	2_Sputum
SAMN54205504	22617X3	22617X3	Mycobacterium tuberculosis	1773	3_Sputum
SAMN54205505	22617X4	22617X4	Mycobacterium tuberculosis	1773	4_Sputum
SAMN54205506	22617X5	22617X5	Mycobacterium tuberculosis	1773	5_Sputum
SAMN54205507	22617X6	22617X6	Mycobacterium tuberculosis	1773	6_Sputum
SAMN54205508	22617X7	22617X7	Mycobacterium tuberculosis	1773	7_Sputum
